# Exploring the gender dimension of problems and needs of patients receiving specialist palliative care in a German palliative care unit - the perspectives of patients and healthcare professionals

**DOI:** 10.1186/s12904-019-0440-7

**Published:** 2019-07-17

**Authors:** Anneke Ullrich, Kristina Grube, Cornelia Hlawatsch, Carsten Bokemeyer, Karin Oechsle

**Affiliations:** Palliative Care Unit, Department of Oncology, Hematology and BMT, University Medical Center Hamburg-Eppendorf, Hubertus Wald University Cancer Center Hamburg, Martinistr. 52, 20246 Hamburg, Germany

**Keywords:** Palliative care, End-of-life care, Gender, Patients, Problems, Needs

## Abstract

**Background:**

Gender disparities of specific symptoms and problems have frequently been observed in palliative care patients, but research rarely focused on the range of problems and needs affected by gender.

**Methods:**

We conducted semi-structured interviews with patients and healthcare professionals (HCPs) of a hospital-based palliative care unit to examine gender effects on patients’ problems and needs based on systematically gathered qualitative data. Content analysis was used to identify emerging themes with data coded using MAXQDA.

**Results:**

Ten patients (5 female, 5 male) and 17 HCPs (12 female, 5 male) were interviewed. Seven categories of gender-specific problems and needs emerged: “physical symptoms, care and body image”, “psychological symptoms and emotional response”, “interaction with the palliative care team”, “use of professional supportive measures”, “activation of informal social networks”, “decision-making”, and “preservation of autonomy and identity”. Both patients and HCPs felt that female patients adopt more expressive coping strategies, have stronger need for communication with and support of HCPs, and activate an extended social network for support and decision-making. Further, both groups thought that male patients mainly rely on social support from partners, have higher expectations to be cared for at home, and have higher need for preservation of autonomy.

**Conclusion:**

Gender relevantly impacts patients’ problems and needs during palliative care. Therefore, gender-sensitive palliative care that acknowledges the patient’s individual situation and respective ramifications are required.

## Background

Gender-sensitivity has become an important goal in preference-based medicine and patient care [1]. Conceptions of gender used in this context often draw on gender as a constructed category, meaning that gender is not an innate category or experience, but “it is something that one *does*, and does recurrently, in interaction with others” [2]. Thus, gender is a construct.

However, systematic data on how gender impacts problems and needs of terminally ill patients in need of palliative care are rare and recommendations for gender-sensitive palliative care are lacking. Some studies suggest only marginal differences in symptom burden between male and female patients with advanced diseases in general, but nausea and emesis [3, 4], pain [4], fatigue [4], and death rattle [5] seem to be more frequent in females. Pain perception seems to be different especially in linking pain with other physical or psychological symptoms [6]. Gender-specific differences might also be relevant for overall symptom distress [3, 4], the combination of synchronous symptoms [6], treatability of symptoms [7], and impact of symptoms on physical functioning and body awareness [8]. Intensity of anxiety, depression, and psychological distress seems to be higher in female patients when they are accompanied by decreased social functioning, limits in pursuing hobbies, increased dependency or disturbed body image [9, 10]. For male, but not female patients with advanced cancer, a correlation of depression and perceived social isolation was demonstrated [8]. Nearing end-of-life, differences in psychological problems and quality of life between men and women seem to decrease [3, 11].

Female patients might accept psychological limitations easier [12] and cope better with loss of bodily autonomy [13]. They were found to earlier accept the incurability of malignant diseases [14] and talk about death and dying [15]. More male than female patients wish intensive care during the last weeks of life [16], but women decide more frequently to undergo aggressive surgery [17]. For continuation of palliative chemotherapies, data are heterogeneous [18, 19]. Gender-related differences have also been found for the trust in accuracy of surrogate decision-making about life-prolonging therapy among older spouses [20].

In the last phase of life, female patients are treated longer and more frequently in hospices or specialist palliative care facilities, while men die more frequently at home, in non-specialist hospitals or nursing homes [21–23]. However, men more commonly express the wish to die at home [24]. Higher healthcare costs at the end of life in female lung cancer patients might reflect the more frequent use of psychosocial support and medical care [25]. Specific supportive treatment, e.g. art or music therapy, are more frequently used by female patients [26, 27]. However, empirical evidence suggests that early integration of palliative care in cancer patients might be of higher impact in male patients [28]. In the course of home-based palliative care, patients who are cared for by female family caregivers receive less additional psychosocial support by other family members or friends [29]. These differences in reality of end-of-life care might also be partly responsible for the higher rates of male patients asking for assisted suicide in countries providing this option legally [30].

Therefore, systematic research is required to recommend gender-sensitive palliative care to equal quality of end-of-life care [31]. A first step in reaching this goal is to identify the range of gender-related issues in the context of palliative care. Thus, an explorative study was undertaken to identify gender-related problems and needs of terminally ill patients receiving specialist palliative care. In order to rely on a broad basis of experiences, the perspectives of both patients and healthcare professionals were included.

## Methods

### Study design

This qualitative study used semi-structured interviews with healthcare professionals (HCPs) and patients of a hospital-based palliative care unit in Hamburg, Germany. The qualitative approach was chosen because it allows participants to set own emphases while not omitting important issues with respect for the claim of systematically gathered qualitative data.

The local ethics committee of the General Medical Council of Hamburg approved the study protocol (reference number 5116). Written informed consent was obtained from all study participants prior to data collection.

### Participants and sampling

A purposive sampling strategy was employed. Within the constraints of availability of participants, willingness to participate, and ability to communicate experiences [32], our strategy aimed at combining typical case sampling with maximum variation sampling (stratified purposive sampling). Stratification criteria were gender and age (both groups), and professional experience (HCPs only).

Eligible participants were patients with incurable, progressive diseases receiving specialist inpatient palliative care. Patients with insufficient knowledge of the German language, aged < 18 years or lacking cognitive capacity were excluded. Patients were recruited at the study ward via a team member introducing the study, with follow up by the researcher. Secondly, HCPs with a minimum professional experience in specialist palliative care of 12 months (doctors, nurses, physiotherapists, psychologists/therapists, pastoral workers) were eligible. HCPs were informed about the study by a senior staff member via email and were asked to contact the research team if they were interested in study participation. A minimum of 20 interviews were planned, but data collection was continued until no new themes emerged [33].

### Data collection

#### Interviews

A semi-structured interview guide was used to explore HCPs’ and patients’ views regarding gender-specific problems (including symptoms) and supportive needs during specialist inpatient palliative care. Guide testing was performed by a group of multidisciplinary HCPs, e.g. to test for comprehensiveness, and two pretest interviews. Pretest interviews were included in the final analyses, since no changes of the interview guide were necessary.

The guide consisted of open questions, permitting the participants to respond in their own words and set their own emphases. After the participants' initial narrative response, we employed prompts to support the interviewees to speak about the research topic in a more structured way [34]. Word-cards visualized a wide range of example areas of problems and needs and were presented simultaneously to avoid possible bias by researcher’s personal priorisation.

A female researcher (KG, medical student), who was neither involved in palliative care nor known by the participants, interviewed all participants after having been trained by AU (female sociologist, highly trained in interviewing). All face-to-face interviews took place on the premises of the palliative care inpatient unit. Each interview was audiotaped with permission and transcribed verbatim.

#### Characteristics and gender-role identity

All participants completed a short questionnaire on sociodemographic, medical (patients) and profession-related (HCPs) variables. Assuming that gender-role identity might influence views and experiences of gender-specific problems and needs, all participants completed the “German Extended Personal Attributes Questionnaire” (GEPAQ) [35], a self-report instrument measuring individual gender-role identity. We used the positive masculinity and femininity scales. Each scale evaluates eight characteristics dyads on a five-point scale, which express socially desired characteristics for masculinity (e.g. independent, active and self-assured) and femininity (emotional, warm-hearted and caring). For each scale, a sum score is calculated (possible range: 8–40) with higher values representing higher expression of masculine or feminine gender-role identity.

### Data analysis

Interview material was iteratively analyzed applying a content analysis approach [36] using techniques of inductive coding. The software program MAXQDA 12 facilitated data management and coding. After reading the interviews, emerging themes were discussed in depth (KG and AU), and a preliminary coding template was developed based on the coding of a subset of five transcripts line by line (KG) and two rounds of feedback within the research team. Text segments were assigned to subcategories, which were than categorized into categories of higher order. This phase included constant comparison to verify and refine early subcategories until no new themes emerged and final categories were conceptualized. To ensure the reliability of the final coding process, both researchers (AU and KG) analyzed the interview material independently. The comparison of the results showed high agreement between the two researchers, and differences were resolved by verbal discussions.

Quantitative data on sociodemographic characteristics and gender-role identity (GEPAQ questionnaire) were analyzed by means of descriptive statistics using IBM SPSS Statistics 22.0.

We used the Consolidated Criteria for Reporting Qualitative Studies (COREQ) framework [37] to report on the design, analysis, and findings of our study where applicable.

## Results

### Characteristics of patients and healthcare professionals

We conducted 27 semi-structured interviews with 17 HCPs (12 female) and 10 patients (5 female). Participant characteristics are presented in Table 1. In both groups, gender-role identity seemed to be balanced in both females and males (Table 2). Mean length of interviews was 36.9 (16–47) minutes for HCPs and 31.7 (20–42) minutes for patients, respectively.

**Table 1:** Characteristics of patients and healthcare professionals

Patients (*N* = 10)	n	%
Sex		
Male	5	50
Female	5	50
Age, mean (range)	61.3	(40–78)
Cancer site		
Gastrointestinal	4	40
Pancreatic	2	20
Lung	1	10
Prostatic	1	10
Mamma	1	10
Malignant melanoma	1	10
Time since first diagnosis in months, mean (range)	34.5	(3–78)
Length of stay at time of interview in days, mean (range)	9.7	(2–23)
HCPs (*N* = 17)	n	%
Sex		
Male	5	29
Female	12	71
Age		
≤ 40 years	6	35
41–50 years	7	41
> 50 years	4	24
Profession		
Physician	4	24
Nurse	7	41
Psychosocial profession	4	24
Pastoral worker	2	12
Work experience in specialist palliative care		
1–5 years	9	53
6–10 years	6	35
> 10 years	2	12
Work experience in specific profession		
1–5 years	2	12
6–10 years	1	6
> 10 years	14	82

*Abbreviations*: *HCPs* healthcare professionals

**Table 2:** Gender-role identity of patients and healthcare professionals

	Patients (*N* = 10)		HCPs (*N* = 17)	
	M (SD)	Range	M (SD)	Range
Whole sample				
Masculinity scale	28.8 (3.5)	25–34	27.3 (3.4)	21–33
Femininity scale	31.2 (3.0)	27–37	33.2 (3.2)	28–39
Male subsample				
Masculinity scale	29.0 (3.3)	25–34	28.8 (4.0)	24–33
Femininity scale	31.2 (2.3)	28–35	33.6 (2.9)	31–38
Female subsample				
Masculinity scale	28.5 (4.2)	24–33	26.7 (3.1)	21–30
Femininity scale	31.3 (4.3)	27–37	33.0 (3.5)	28–39

Higher values reflect higher expression of masculine or feminine gender-role identity

*Abbreviations*: *HCPs* healthcare professionals, *M* Mean, *SD* standard deviation

### Categories developed: gender-specific problems and needs

The analysis of the interview material revealed seven main categories of gender-specific problems and needs: “physical symptoms, care and body image”, “psychological symptoms and emotional response”, “interaction with the palliative care team”, “use of professional supportive measures”, “activation of informal social networks”, “decision-making”, and “preservation of autonomy and identity”. Table 3 presents the seven categories of gender specific-problems and needs, its subcategories and the number of patients and HCPs describing gender-specificity (yes or no), thus adding a quantitative element to the findings.

**Table 3:** Gender-specificity in patients’ problems and needs: overview of categories and subcategories

Category	Subcategories	Gender-specificity^a^					
		Patients (*N* = 10)			HCPs (*N* = 17)		
		N	Yes (n)	No (n)	N	Yes (n)	No (n)
1. Physical symptoms, care and body image	1.1 Physical symptoms	8	5	3	11	4	7
	1.2 Coping with physical symptoms	2	2	0	14	14	0
	1.3 Body image and appearance	2	2	0	5	4	1
	1.4 Preference for gender-sensitive physical nursing	10	7	3	10	8	2
2. Psychological symptoms and emotional response	2.1 Psychological symptoms	6	4	2	10	5	5
	2.2 Coping with psychological symptoms	7	7	0	13	10	3
3. Interaction with the palliative care team	3.1 Need for communication with the team	9	8	1	18	16	2
	3.2 Occasions and themes of conversation	2	0	2	5	4	1
	3.3 Choice of contact persons and confidents	4	3	1	12	8	4
	3.4 Appreciation of authority and professional expertise	1	1	0	8	7	1
	3.5 Trust and cooperation	7	3	4	9	5	4
4. Use of professional supportive measures	4.1 Active demand for support	3	2	1	7	7	0
	4.2 Actual utilisation of support	7	5	2	21	20	1
5. Activation of informal social networks	5.1 Delegation of responsibilities and tasks	8	3	5	18	16	2
	5.2 Maintaining social relationships	4	3	1	9	6	3
	5.3 Care for the dying	4	2	2	6	3	3
6. Decision-making	6.1 Course of decision-making	6	5	1	1	1	0
	6.2 Involvement of significant others	1	1	0	7	5	2
	6.3 Decisional basis for or against home-based care	4	4	0	10	9	1
	6.4 Kind of further care	6	4	2	13	8	5
	6.5 End-of-life treatment and wishes	4	1	3	9	1	8
7. Preservation of autonomy and identity	7.1 Need for preserving autonomy and control	10	9	1	14	10	4
	7.2 Need for maintaining one’s identity	10	9	1	10	10	0

*Abbreviations*: *HCP*s healthcare professionals

^a^*N*, Number of patient or HCP interviews in which the subcategory was coded; *Yes (n)*, number of interviews in which the patient or HCP described experience of gender-specificity; *No (n)*, number of interviews in which the patient or HCP described experience of no gender-specificity

#### Category 1: Physical symptoms, care and body image

Respondents from both groups were rather indifferent regarding gendered disparities in *physical symptoms*. Only two patients referred to *coping with physical symptoms*, while HCPs experiences showed a clear tendency of gender-specific coping: Respondents noted more inexpressive coping responses (e.g. enduring symptoms) in male patients, whereas women were thought to be more expressive (e.g. open disclosure of symptom burden, active seeking for help). As one female HCP said:

*“I think men call later than women when suffering from symptoms like nausea. They seem to feel that they have to endure symptoms as long as possible.” (H07, 44)*

*Changes of body image and appearance* were thought to be of more emotional impact in females, who were attributed having higher sense of shame and concerns about loss of attractiveness. As one female HCP narrated:

“*I think men pay less attention to it. […]. Women are more attentive to their ideal: what do I look like, I have to – want to – care for myself, then I feel better.” (H04, 38)*

Regarding intimate care (e.g. bathing, toileting), particularly female and migrant patients were reported by HCP to have *preferences for gender-sensitive physical nursing*. Among patients, most experienced comfort by gender-sensitive physical nursing.

#### Category 2: Psychological symptoms and emotional response

Both patients and HCPs were ambivalent regarding gender-differences in *psychological symptoms,* as one female HCP said:

*“It is a whole palette I see. But I could not say they are gendered. Grief – I’ve seen men crying and women saying ‘we’ll make it’.” (H12, 6)*

Narrations of both groups showed gender-specificity of *coping with psychological symptoms*. Patients and HCPs ascribed female patients a more problem-focused coping, such as seeking for help, whereas avoidance and disengagement was often mentioned related to males. Acceptance of the emotional response was also primarily attributed to female patients, as one male patient pointed out:

*“I think they [women] are more likely to admit it, ‘I have a depression right now.’ I think it is easier for women to acknowledge depressive feelings.” (P08, 40)*

#### Category 3: Interaction with the palliative care team

Patients and HCPs alike addressed gender-specific *needs for communication with the team* with more frequent and intimate conversations being related to female patients. A male patient narrated male communicative behavior to be more controlled:

*“Do I communicate about it or not?’ I believe women communicate more openly.” (P08, 41)*

Regarding *occasions and themes of conversation*, both groups viewed men to initiate conversations on a more target-oriented basis, as pointed out by a female HCP:

*“In my view, male communication […] often starts with a clear need.” (H17, 12)*

Female patients were felt to be more likely to include emotional and existential themes in their communication. In *choice of contact persons and confidents*, both women and men were reported to prefer HCPs of the same sex, as one female HCP narrated:

*“I also believe that for some [patients] it feels better if a man enters the room…or a woman. Some just cannot connect with people of one sex or the other.” (H04, 49)*

On the other hand, this phenomenon was perceived to disappear as patient-team relationship deepened. Cases of lacking *appreciation of authority and professional expertise* in male patient/female HCP constellations were mentioned by both patients and HCPs, with the patient’s socialization (cultural/religious, generation) playing a major role. As one male patient said:

*“I think, men tend to believe and accept more what is said by a male doctor.” (P01, 18)*

This was affirmed by a female HCP:

*“I feel that they [men] are more reluctant to take advice from a women than a man, specifically male patients from specific cultural backgrounds.” (H07, 57)*

Respondents were ambivalent whether *trust and cooperation* are gender-specific: Some felt that female patients were more cooperative in finding problem-oriented solutions and showed higher trust in HCPs advice. Others felt that trust is less a question of gender but of character and personal experiences.

#### Category 4: Use of professional supportive measures

*Active demand for support*, particularly in terms of psychological services, was more often ascribed to female patients, and one female patient narrated:

*“I think that women more often say what they want or don’t want. They handle their disease and needs with more openness. Women rather than men tell that they want to talk to someone or that they wish to take part in supportive interventions, such as art or music therapy.” (P07, 6)*

Regarding *actual utilization of support*, men’s use of psychological services was perceived to be less frequent both among patients and HCPs, even when actively offered by team members. On the other hand they were described to be particularly interested in measures targeting physical activity and strength. Many respondents agreed that men’s preference of not wanting to share feelings does not mean that they do not need support. Further, a possible relation between utilization patterns and specialist psychosocial offers being a female domain was questioned by one male HCP:

“*In my perception, women accept those [psychosocial support interventions] much better, not all women, but with consistence. Music therapy, art therapy, psychosocial care…. Maybe this also arises from the fact that supportive measures are often offered by women.” (H10, 24)*

#### Category 5: Activation of informal social networks

Many patients as well as HCPs proposed that the *delegation of responsibilities and tasks* is gender-specific. In this context, gender did not necessarily affect delegation itself, but objectives of delegation, as one male patient pointed out:

*“It is hard for me to adapt to the physical symptoms I experience. I always said, I will do my stuff whatever happens. That’s how I am, how many men are. It was hard for me to realize that I can’t 'pick the peas' myself anymore.” (P02, 56)*

In HCPs views, it is particularly difficult for male patients to delegate finances and legal matters, whereas (particularly older) women rather worry about transferring domestic and caring tasks. In addition, female patients were reported *maintaining social relationships* with an extended network of family and friends throughout palliative care, whereas male patients tend to have fewer confidents. Mainly, respondents felt that male patients relied on their spouse/partner, and that relational closeness in respective relationships often increased over time. Respondents noted that activation of social networks is also impacted by cultural influences and level of family cohesion. Regarding *care for the dying*, female patients more often activated extended social networks for emotional and practical support, for both themselves and their family caregivers. One male patient connected this to feelings of burdening the family, as he highlighted

*“that women are indeed more concerned about how caregiving will affect their family. That they don’t want to be a burden to them.” (P01, 24)*

#### Category 6: Decision-making

Regarding the *course of decision-making*, female patients were identified to prefer a more active role. Also, *involvement of significant others* was felt to be more common in female decision-making: while these often include an extended network of family and friends, male patients predominantly rely on spouses/partners. As one female HCP pointed out:

*“Women more often seek for counselling than men do. Men rather decide on their own, while women take the decision in partnership with their family and the team.” (H10, 27)*

Respondents were ambivalent on whether the *kind of further care* is gendered: Some thought that male patients more often received home-based care, others narrated no gender-differences. As one female HCP expressed,

*“I think both men and women want to go home. Also, families wish to take patients home so that the patient does not think to be pushed away.” (H12, 24)*

A male patient complemented:

*“One is so familiar with home…who wants to go? Rather, one lets someone into home to help caring for the patient.” (P06, 52)*

In contrast, across both patients and HCPs, the *decisional basis for or against home-based care* was seen to depend on the patient’s gender. In male patients, the patient himself and his network more often expect partners to take the caregiving role, while female patients often worried about being a burden to others or were not confident in the partner’s resources for providing home-based care, as was described by one male patient:

*“I think that men are more often demanding it and put pressure on women: ‘You always have cared for me, now go on with caring for me.’ And women rather say ‘He will not make it on his own.’”.(P03, 51)*

Only few respondents considered *end-of-life treatment and wishes* (e.g. withdrawal of palliative chemotherapy, antibiotics) to be gender-specific, and one male patient narrated:

*“Treatment at end of life… Is there pain? Is all hope for cure gone? If yes, I think men and women just want the same: to die without pain”. (P08, 47)*

#### Category 7: Preservation of autonomy and identity

Patients and HCPs agreed on gender-differences regarding patient’s *need for preserving autonomy and control*. Men were thought to have stronger need for autonomy and control, as one male patient pointed out:

*“I have a strong need for autonomy… I am very sensitive if someone interferes in things, which are not his business and which I have thought about thoroughly. I want to be the one saying ‘I need help’.” (P03, 28)*

In addition, male patients were perceived to have a stronger *need for maintaining one’s identity*, which respondents often understood as recapturing a past self in order to preserve a current self. This included role erosions (e.g. family role, being an employer) and activities (e.g. hobbies). Overall, male patients were felt to suffer more from being dependent on others and the idea of diminished social value. One female HCP connected this with feelings of powerlessness:

*“In case of a terminal illness men feel more helpless and without power because they can’t continue their living habits and fulfil their social roles, they lose ability to steer decisions. We then often notice a certain… not anger, but struggle with fate.” (H02, 56)*

### Awareness of underlying normative ideas

Beyond the seven categories of gendered problems and needs, an eighth category emerged, namely “awareness of underlying normative ideas”. During the interviews, respondents often addressed normative ideas of gender roles and the social and cultural construction of gender. For example, the idea that men urge to maintain a masculine image dictating that men should not show weakness or emotional strain. In the context of trust and cooperation, one patient connected males’ behavior to masculinity, as he pointed out:

*“Excluding the character of a person, I think that women are more cooperative than men are. Because in a man, due to genetic reasons, alpha behavior might come up, to be the lord: ‘I am the boss and I have a problem to subordinate’.” (P08, 29)*

Many respondents of both groups thought that women might have higher ability to acknowledge psychosocial burden and might be less concerned about possible stigmatization, e.g. resulting from psychological treatment. Another frequent explanation for gendered disparities was that, over the life span, women more often cultivated to talk about their emotional problems and needs. Gendered expectations about nurturing and caring were highlighted in almost all interviews.

## Discussion

This qualitative interview study revealed patients’ and HCP’s views on the impact of gender on problems and needs of patients receiving specialist inpatient palliative care. Based on content analysis of 27 interviews, we found that respondents described a wide range of gender-specific problems and needs, reflected in seven categories. This provides new and in-depth insights on how gender might shape patient’s coping responses to physical and psychological symptoms, caring for and interacting with the patient, patient’s social resources, and ways of decision-making.

Patients receiving specialist palliative care have complex and changing physical as well as psychosocial problems and needs along their disease trajectory. Existing research shows gender-disparities in symptom burden [3–6], but until today, there is little systematic evidence. In addition, differences in psychological problems and quality of life seem to be time-dependent [3, 11]. The heterogeneity of findings was also reflected in our results with the scope or intensity of symptoms not being clearly identified to be gender-specific. In contrast, our study suggests that gender is perceived to affect coping responses to physical and psychological burden during palliative care. Female patients were described to use more expressive strategies to respond to their illness, e.g. sharing feelings, and to have more abilities to acknowledge their situation. Correspondingly, in a study focusing on gender differences in the evolution of illness understanding, female patients showed higher levels of terminal illness acknowledgment [14]. Gender-related coping strategies have important implications on how to engage female and male patients in the disease process and how to support their coping abilities. Neglect of gender-differences might prevent patients from finding their subjective way of coping with their illness.

Regarding interaction with the palliative care team, our results indicate that female patients might need intensified communicative attentiveness. Women were more likely to include emotional and existential themes in their communication, including death and dying. In a study on gender differences in talking with chaplains about impending death, men showed more resentments related to death talk than women did: within the first contact, 80% of female compared to 30% of male patients spontaneously raised the topic of their own death. Overall, 9% of women and 37% of men did not want to talk about their own imminent dying at all [15]. As sharing feelings and communication about troubling issues might be a rather feminine perspective, male gender scripts of communication need to be acknowledged. For example, meeting a target-oriented communication style might include focusing on problem identification or coming up with an action plan rather than talking about emotions. Our study also suggested gendered disparities regarding the interaction with informal caregivers. We found that male patients were perceived to rely on smaller social networks, whereas female patients rely on extended networks which they actively use.

Gender schema theorists have proposed that, throughout life, people are taught which behaviors are desirable for each gender in society [38]. Women are positioned as natural emotional carers of family members [39], and studies show that caregiving in palliative care continues to be a female domain [40, 41]. Our study showed that spouses/partners of male patients are more often expected to allow for home-based care and female patients are ascribed higher worries to be a burden to others. In consequence, there was a tendency in our results that the decisional question of place of further care might be gendered, with male patients receiving more home-based care. Indeed, previous studies reported that women are more likely to stay longer inside the purview of formal palliative care and receive less informal care, with reluctance to be a burden and outliving male spouses/partners being possible explanatory factors [21, 22, 42].

In our study, male patients were thought to be more passive in decision-making and tended to mostly incorporate spouses/partners into the decision-making process. Their female counterparts were identified to prefer an active role and the inclusion of supportive persons in decision-making. Most respondents noted no gender-differences in therapy-related decisions, while previous research did. Studies showed men to be more likely to receive aggressive, non-beneficial intensive care near death than women did [16], and women to be more likely to undergo aggressive surgery [17]. Regarding issues of autonomy and self-reliance, we found higher needs of preserving autonomy and identity in male patients. This finding corresponds to typically male socialization teaching men to value independence and autonomy, while female socialization is rather based on values such as connectedness and supportiveness [43]. In our study, respondents felt that depending on others is more stressful and burdening for men compared to women, and one study found feelings of dependence to be associated with higher burden of depression in men [7]. Safeguarding patients’ autonomy is a key objective of clinical practice in palliative care. Gender-sensitive approaches of preserving autonomy should consider predominant values (e.g. self-reliance, supportiveness) and problems (e.g. undermined dependency), possibly rooted in female and male socialization, and thus socially mediated.

### Strengths and limitations of the study

We used a qualitative approach to explore gender-differences in palliative care representing a poorly researched topic with lacking systematic data. The perspectives of both patients and HCP’s were included respecting the crucial fact that gender-sensitive palliative care is grounded in what involved parties consider to be gendered problems and needs. In both groups, we used purposive sampling to collect perceptions and experiences of persons with differences in age, gender, disease trajectory (patients), and profession (HCPs).

It has to be noted, that the results of this study are subject to certain limitations. First, all patients and HCPs were recruited from a single institution. Therefore, shared experiences and normative ideas of HCPs, but also patients, could shape our results in terms of generizability. Gender-related experiences may differ, for example, in more rural or urban areas as well as cross-culturally. Second, there may be a social desirability response bias, as patients and HCPs’ narrations may include attempts to construct favorable images of themselves and answer in a socially desirable way. Third, although self-report is valuable to describe empirical aspects of palliative care, we have to be aware that respondents have limited capacity to reflect on their inner life [44]. Own expectations on how men or women should react in relation to their gender might occur in a “black box” [44] of respondents awareness and is likely to influence their views. On the other hand, patients and HCPs showed rather balanced gender-role identity and actively reflected on normative ideas of gender roles during the interviews. Fourth, the sample size of patients in this study is small and not balanced to the number of interviews conducted with HCPs. Therefore, findings from this study might underestimate gender-differences from the patient’s point of view. However, in our study data saturation was reached, suggesting that the overall sample size was sufficient. Future research might examine the perspectives of a larger range of patients across different institutions providing palliative care.

## Conclusions

In conclusion, we have demonstrated that the multifaceted problems and needs of patients receiving specialist palliative care are closely tied to gender-related expectations and experiences. This has considerable consequences for the provision of palliative care, in terms of paying closer attention to the gender dimension and promoting HCPs’ skills in gender-sensitive care. It is important to develop a better understanding of gender-specific needs, thus contributing to an improved quality of care and well-being of the patient. HCPs need to be aware of the importance of taking into account the extent and particularities of gender-related issues. Strategies should be implemented to support HCPs in providing gender-sensitive palliative care, including the development of clinical guidelines. Likewise, although some research has examined gender-related issues in palliative care, lacking systematic data does not allow for clinical recommendations. The results of this study suggest a wide range of gender-specific problems and needs, and future research could help to gain a better picture on how gender shapes palliative care, how gender-sensitive care could be fostered, and also to help design adequate trainings for HCPs in how to implement gender-sensitive care.

## Data Availability

The authors have full control over the primary data. The data analyzed in this study are housed at the Palliative Care Unit, Department of Oncology, Hematology and BMT, University Medical Center Hamburg-Eppendorf, Martinistrasse 52, 20246 Hamburg, Germany. As per the ethical committee approval, this dataset is subject to ethical restrictions, and informed written consent of study participants does not include publication of raw data in terms of interview manuscripts. All relevant data for the conclusions are presented in the manuscript.

## Abbreviations

GEPAQ: German Extended Personal Attributes Questionnaire
HCPs: Healthcare Professionals
